# Cancer-derived small extracellular vesicles: emerging biomarkers and therapies for pancreatic ductal adenocarcinoma diagnosis/prognosis and treatment

**DOI:** 10.1186/s12951-022-01641-0

**Published:** 2022-10-14

**Authors:** Wei Zhang, Douglas H. Campbell, Bradley J. Walsh, Nicolle H. Packer, Dingbin Liu, Yuling Wang

**Affiliations:** 1 School of Natural Sciences, Faculty of Science and Engineering, ARC Centre of Excellence for Nanoscale BioPhotonics (CNBP), Macquarie University, 2109 Sydney, NSW Australia; 2 Minomic International Ltd, Macquarie Park, 2113 Sydney, NSW Australia; 3 State Key Laboratory of Medicinal Chemical Biology, Research Center for Analytical Sciences, and Tianjin Key Laboratory of Molecular Recognition and Biosensing, College of Chemistry, Nankai University, 300071 Tianjin, China

**Keywords:** Pancreatic cancer, Extracellular vesicles, Cancer diagnosis/prognosis, Cancer treatment

## Abstract

Pancreatic ductal adenocarcinoma (PDAC) is one of the most fatal cancers worldwide with high mortality, which is mainly due to the lack of reliable biomarkers for PDAC diagnosis/prognosis in the early stages and effective therapeutic strategies for the treatment. Cancer-derived small extracellular vesicles (sEVs), which carry various messages and signal biomolecules (e.g. RNAs, DNAs, proteins, lipids, and glycans) to constitute the key features (e.g. genetic and phenotypic status) of cancer cells, are regarded as highly competitive non-invasive biomarkers for PDAC diagnosis/prognosis. Additionally, new insights on the biogenesis and molecular functions of cancer-derived sEVs pave the way for novel therapeutic strategies based on cancer-derived sEVs for PDAC treatment such as inhibition of the formation or secretion of cancer-derived sEVs, using cancer-derived sEVs as drug carriers and for immunotherapy. This review provides a comprehensive overview of the most recent scientific and clinical research on the discovery and involvement of key molecules in cancer-derived sEVs for PDAC diagnosis/prognosis and strategies using cancer-derived sEVs for PDAC treatment. The current limitations and emerging trends toward clinical application of cancer-derived sEVs in PDAC diagnosis/prognosis and treatment have also been discussed.

## Introduction

Pancreatic ductal adenocarcinoma (PDAC), accounting for more than 90% of pancreatic cancers, is one of the most aggressive malignancies with a 5-year survival rate of 8-9%.[1–6] It has been reported that only 15–20% of PDAC patients can be surgically resected; the other 80–85% of patients present with unresectable metastatic or locally progressed diseases.[7, 8] Most PDAC patients still suffer local recurrence or systematic metastasis in 12 months after surgery, with an overall 5-year survival rate between 20% and 30%.[9] There were over 55,989 new cases (29,673 men and 26,316 women) of PDAC diagnosed in the United States in 2022 according to cancer statistics of American Cancer Society.[10] And PDAC has been estimated to surpass colon cancer as the second leading cause of cancer-related death in the United States by 2030.[11] The majority of patients failed to be diagnosed in the early stage of PDAC, which caused approximately 48,220 deaths.[12] Despite various factors that influence cancer patient outcomes, there remain two essential problems, namely reliable PDAC diagnosis/prognosis and effective treatment regimes. The poor diagnosis/prognosis of PDAC is attributed to multiple reasons, including non-specific symptoms or even no symptoms in the early stage of PDAC, lack of sensitive and specific PDAC biomarkers, and difficulties in imaging early-stage tumors.[5] While the treatment failure of PDAC is typically caused by its insidious onset, high invasiveness and metastasis[13], detecting PDAC at an early stage is crucial to improve the therapeutic effect and thereby significantly increase the overall survival of PDAC patients.

Currently, there are no validated and specific tests to reliably diagnose PDAC in clinic, particularly during early stages. PDAC is usually diagnosed by biochemical examination, imaging examination and tissue biopsy.[14] The most extensively evaluated biomarker for biochemical examination of PDAC is carbohydrate antigen 19−9 (CA19-9). However, CA19-9 has insufficient sensitivity and specificity to distinguish the PDAC patients from healthy people or patients with other pancreatic disease (chronic pancreatitis, acute pancreatitis, etc.).[15, 16] Studies have shown that multiple biomarkers provide more accurate results than individual biomarkers.[17, 18] For example, Shreya et al. identified a diagnostic panel of 4 serum biomarkers (S100A2, A100A4, CA-125 and CA19-9) which had higher diagnostic potential (AUC 0.913) than CA19-9 alone (AUC 0.869) in a small study of 120 PDAC patients and 80 healthy controls.[19] However, larger clinical trials are still essential to validate its accuracy and investigate the potential for early PDAC detection. Imaging examination, such as contrast-enhanced computed tomography (CT), magnetic resonance imaging (MRI), and endoscopic ultrasound are quite expensive and inefficient to detect early lesions or to differentiate benign from malignant lesions.[20] On the other hand, tissue biopsies are invasive, show low sensitivity and require specialized surgical skills and facilities for sampling.[21, 22] Therefore, PDAC patients can only gain very limited benefit from the advanced surgical techniques, perioperative management and oncological treatments due to the weaknesses of the current diagnostic methods for early PDAC diagnosis. There is an urgent need for reliable, specific and sensitive PDAC biomarkers and diagnosis methods to improve the diagnostic accuracy of PDAC at early stages.

Earlier diagnosis of cancer would give patients more time for treatment, but patient outcomes will not be significantly improved without efficient treatment plans. The currently available therapeutic options for PDAC involve the combination of chemotherapy, surgery, radiation and immunotherapy, most of which are palliative treatments aiming to relieve the symptoms and prolong the patient survival rate.[23] However, PDAC can survive under these harsh conditions and increase proliferative ability because of its genetic and metabolic remodeling.[24, 25] Furthermore, a dense and diffuse stroma forming around the tumor can increase its resistance to treatments and affect the tumor progression.[25] PDAC may also develop chemoresistance during treatment due to tumor heterogeneity and plasticity.[26, 27] These characteristics make PDAC resistant to traditional treatment approaches and lead to poor clinical outcomes, thus innovative therapies are required to improve the prospects of PDAC patients. Fortunately, researchers have discovered emerging biomarkers and treatment candidates for both PDAC diagnosis and therapy after intensive studies of extracellular vesicles (EVs) in recent years.

**Fig. 1:**
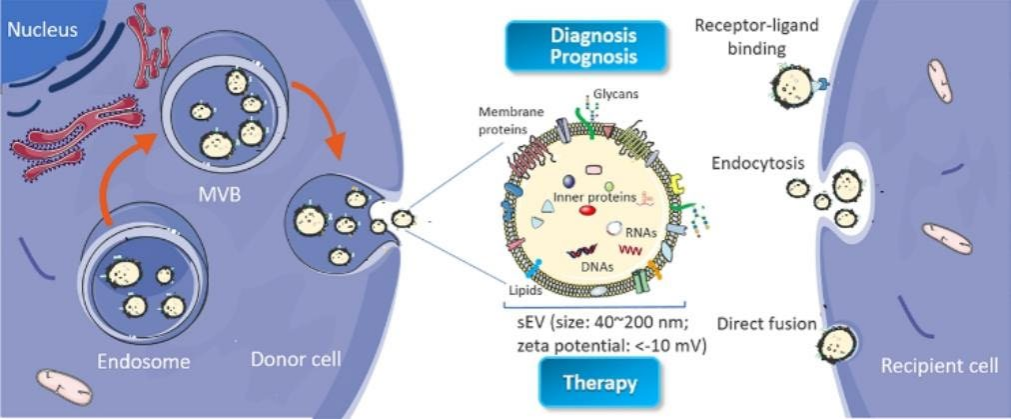
Biogenesis and identification of small extracellular vesicles (sEVs). sEVs originate from the endosomal pathway by the formation of endosomes and MVBs. When MVBs fuse with cell membrane, sEVs are released into extracellular milieu. sEVs are composed of a lipid bilayer vesicle containing nucleic acids, proteins, lipids, glycans, and other small molecules

EVs released from a variety of cell types are classified into three broad groups according to their size, pathway of origin, and biomolecules: small EVs (sEVs) (namely exosomes, 40–200nm) (Fig.1), microvesicles (microparticles or ectosomes, 50-2000nm) and apoptotic bodies (500–4000nm).[28, 29] In the past decade, sEVs have attracted worldwide attention among researchers from various fields of life sciences because of their special and important roles in various biological functions (angiogenesis, cell apoptosis, inflammation and immune regulation, etc.) at normal physiological condition as well as pathological condition, determining by which cells they originate as well as the status of these cells at time of sEV generation.[30] sEVs derived from cancer cells played a crucial role in PDAC biology, including tumorigenesis, cancer progression, cancer metastasis, immune regulation and therapeutic resistance, etc., showing great value in cancer studies.[31, 32] Cancer-derived sEVs are small, lipid bilayer membrane vesicles generated inside the cell in multivesicular bodies (MVBs), which release cancer-derived sEVs into the extracellular microenvironment by fusion with the cell membrane (Fig.1).[1, 33] These cancer-derived small vesicles contain numerous biomolecules including DNAs, RNAs, proteins, glycans and lipids, which can be transported from donor cells to other recipient cells (adjacent or distant cells) mainly by receptor-ligand binding, endocytosis and direct fusion, to establish a desired small-scale environment for modifying the functions (gene expression, signaling, and overall functions) in states of cancers.[34] Although the mechanism of cancer-derived sEV for tumorigenesis is complex, it is generally accepted that the interactions of cancer associated proteins and oncogenes between cancer cells and healthy cells promote the process. [35] These cancer associated biomolecules in cancer derived sEVs can activate the signal transduction pathways and induce cellular change within recipient cells to regulate cancer growth and metastasis.[36] Body fluids such as blood in cancer patients contain diverse mixture of EV subsets, among which cancer-derived sEVs (~ 23–66% of total sEVs in plasma) are substantial and important EV subset, acting as an indicator of tumor and holding a significant potential to serve as a liquid biopsy tool for cancer diagnosis/prognosis.[37–40] These sEVs secreted by cancer cells can be collected and provide the dynamic information from the tumors at the time of blood drawing. Thus, cancer-derived sEVs are promising cancer biomarkers for non-invasive cancer diagnosis/prognosis.[41–44] In addition, cancer-derived sEVs have also been explored for their use in cancer therapeutics. Using sEVs in therapeutics has been studied for preventing the formation and release of cancer-derived sEVs; using cancer-derived sEVs as drug delivery vesicles; as well as using cancer-derived sEVs in immunotherapy. Compared with synthetic nanoparticles, cancer-derived sEVs encompass several desirable attributes: intrinsic ability to carry biomolecules such as RNAs, DNAs, and proteins ; immune tolerance when using autologous-derived sEVs; desirable stability in body fluids; natural targeting property of cancer cells; ease of surface modification and ability to cross biological barriers, such as the blood-brain barrier (BBB).[45–47] Overall, these advantageous features allow cancer-derived sEVs to be promising candidates for providing novel therapeutic strategies for cancer treatment.

Cancer-derived sEVs have been reported to play different roles in PDAC, including cancer initiation, progression, metastasis, drug resistance, cancer diagnosis/prognosis and treatment. [32, 35, 48] In this review, we will mainly focus on the most recent progress in the use of key molecules of cancer-derived sEVs as emerging biomarkers for PDAC diagnosis/prognosis and cancer-derived sEVs based therapeutic strategies for PDAC treatment, as well as discussing the current hurdles and perspectives for further clinical applications, with the aim of gaining new insights for researchers working on sEVs in cancer diagnosis/prognosis and treatment.

## PDAC diagnosis/prognosis

**Fig. 2:**
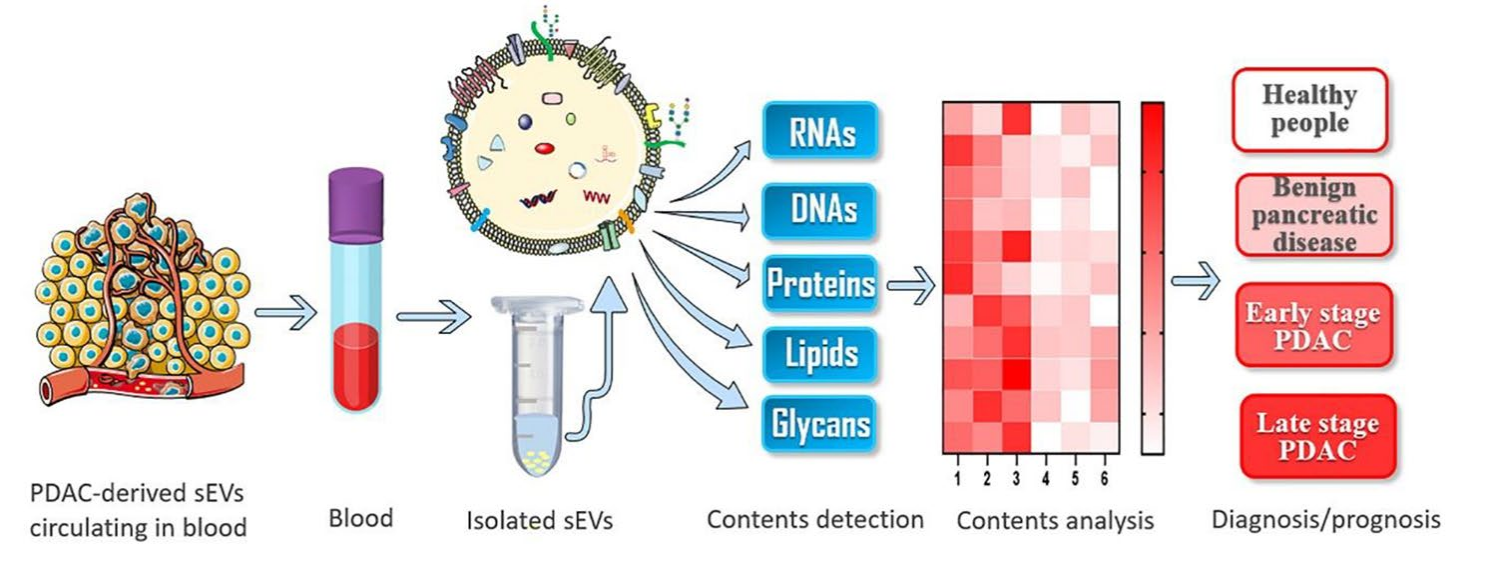
Molecular analysis of components in/on sEVs for PDAC diagnosis/prognosis. PDAC-derived sEVs circulating in blood can be enriched by techniques such as ultracentrifugation. Molecular components including RNAs, DNAs, proteins, lipids and glycans can be analyzed to generate the unique molecular signature for PDAC diagnosis/prognosis

Given the absence of non-invasive and robust biomarkers for PDAC diagnosis, there has recently been significant interest in the use of PDAC-derived sEVs as biomarkers due to their diverse molecular contents. The biogenesis of sEVs enables the packing of these complex extracellular and intracellular molecular contents into sEVs (or on the surface of sEVs) in a cell specific manner.[49] These molecular contents can reflect the key features of cells from which they originate.[50] As the contents of PDAC-derived sEVs are cell-type specific, PDAC-derived sEVs may provide a unique ‘signature’ of genetic and phenotypic status of the tumor.[51] This molecular signature is able to discriminate cancer-derived sEVs from different types of cancer cells, as well as distinguish cancer-derived sEVs from healthy sEVs. Cancer-derived sEVs also carry specific oncogenes and oncoproteins (mutant KRAS, etc.), which can be used to detect cancer-derived sEVs from other sources of sEVs as well.[52] Moreover, sEVs secreted by PDAC cells can be easily collected from body fluids, such as blood. Under the protection of the endogenous membrane of the sEVs, these biomolecules carried by sEVs can remain stable inside the blood circulation, which makes the PDAC diagnosis/prognosis more reliable. PDAC-derived sEVs in blood can be enriched using different isolation approaches (e.g. ultracentrifugation, immunoaffinity isolation, polymeric precipitation isolation and size exclusion chromatography) and their molecular components (e.g. RNAs, DNAs, proteins, lipids and glycans) can be analyzed by corresponding techniques (e.g. polymerase chain reaction, gel electrophoresis, flow cytometry and mass spectroscopy) for PDAC diagnosis/prognosis (Fig.2). There are plenty of excellent review articles [30, 31, 49, 53] on sEVs’ origin, isolation, characterization and analysis techniques, which are not the focus of this review article. Here we will initially focus on the key molecules that are carried by PDAC-derived sEVs and discuss their potential as biomarkers in PDAC diagnosis/prognosis .

**Fig. 3:**
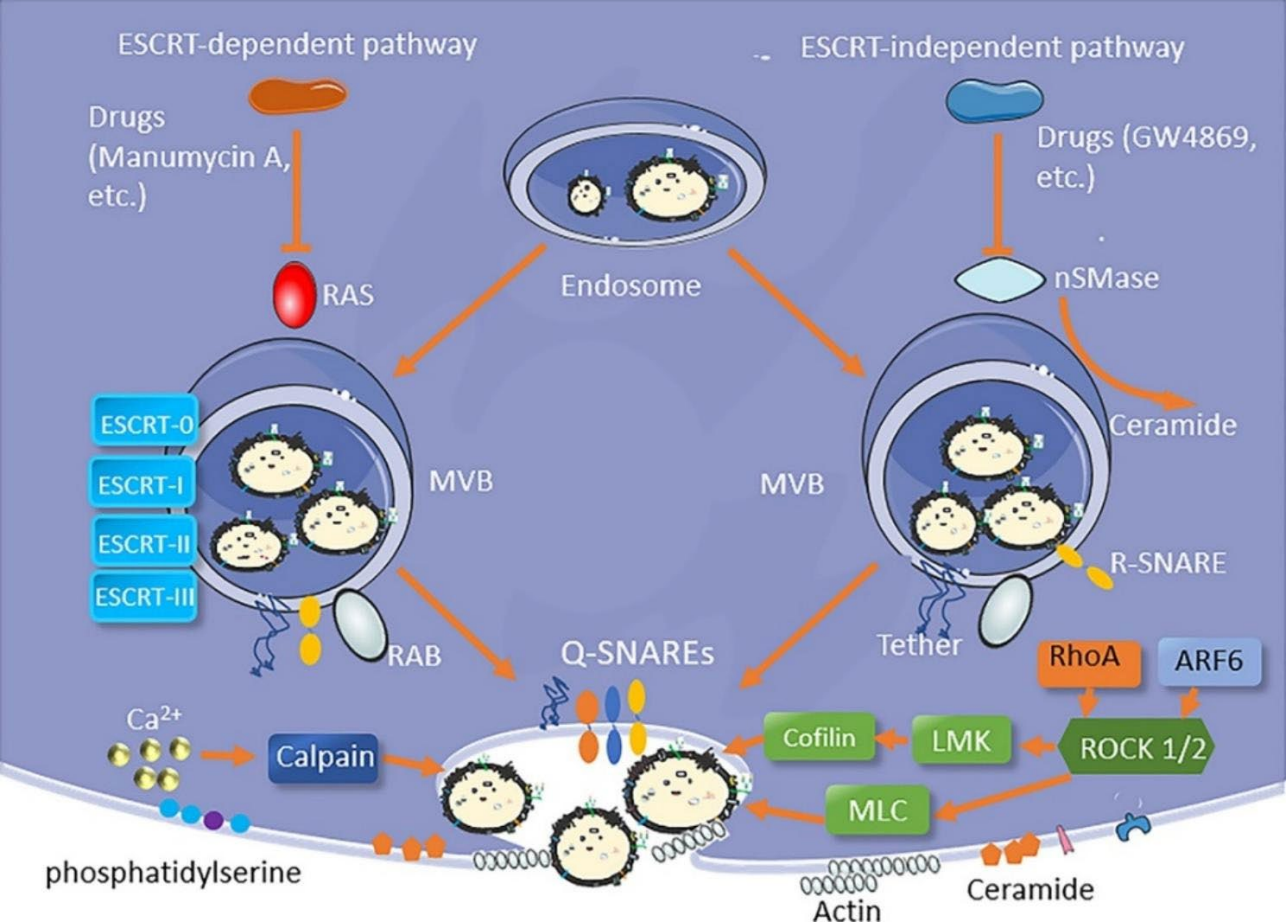
Schematic of the formation and release of sEVs. MVB biogenesis is associated with ESCRT-dependent and ESCRT-independent pathways, agents such as ROCK, RAB, SNARE, Ca^2+^ affect the release of sEVs from cells

### RNAs

PDAC-derived sEVs contain different forms of RNAs, including micro RNAs (miRNAs), messenger RNAs (mRNAs), long non-coding RNAs (lncRNAs) and circular RNAs (circRNAs).[54, 55] RNAs in sEVs have been extensively studied, due to their critical roles in regulating almost all aspects of cancer-related metabolism and function.[56] Among these RNAs, miRNAs are the most studied target for cancer diagnosis/prognosis, whereas other RNAs may also act as potential biomarkers for PDAC diagnosis/prognosis. [57] Table1 provides the reported RNA biomarkers for PDAC diagnosis/prognosis.

**Table 1:** sEV RNA biomarkers for PDAC diagnosis/prognosis

RNA types		Biomarkers	Sources	Patient numbers	Discoveries and diagnostic performance	Ref.
miRNA		miR-196a	Plasma	Stage I-IIA n = 15	Higher miR-196a expression in sEVs from PDAC patients with AUC of 0.81	[58]
		miR-10b	Plasma	N = 3	The expression of miR-10b was significantly higher in sEVs from PDAC patients when compared with patients with chronic pancreatitis (CP) or normal controls	[59]
		miR-451a	Plasma	Stage I n = 7, stage II n = 43	The level of miR-451a showed a significant association with cancer diagnosis and cancer stage discrimination (stage I vs. healthy volunteers P= 0.019, stage II vs. healthy volunteers P< 0.001, stage II vs. stage I P= 0.041)	[60]
		miR-125b-3p, miR-122-5p, and miR-205-5p	Plasma	N = 65	MiR-125b-3p, miR-122-5p, and miR-205-5p were overexpressed in PDAC patients than healthy people with AUC values of 0.782, 0.814, and 0.857, respectively	[61]
		miR-10b, miR-21, miR-30c, miR-181a and miR-let7a	Plasma	N = 29	High levels of miR-10b, miR-21, miR-30c, and miR-181a and a low level of miR-let7a in sEVs could differentiate PDAC from normal control and CP samples with AUC of 1.00	[13]
		miR-1226-3p	Serum	N = 17	The expression of miR-1226-3p was downregulated in PDAC patients compared to benign pancreatic lesions with AUC of 0.74	[62]
		miR-17-5p and miR-21	Serum	N = 22	High expression of miR-17-5p and miR-21 in sEVs from PDAC patients with AUC of 0.887 and 0.897, respectively	[63]
		miR-451 and miR-720	Serum	N = 52	PDAC patients had significantly higher levels of miR-451 and lower levels of miR-720 in sEVs than healthy controls with AUC of 0.93 and 1.00, respectively	[64]
		miR-191, miR-21 and miR-451a	Serum	N = 32	The expression of miR-191, miR-21 and miR-451a in sEVs was significantly up-regulated in patients with pancreatic cancer and IPMN compared to controls with AUC of 0.788, 0.826 and 0.759, respectively	[65]
		miR-1246, miR-4644, miR-3976 and miR-4306	Serum	N = 131	The level of miR-1246, miR-4644, miR-3976 and miR-4306 were significantly upregulated in 83% of the cancer patient group, but rarely in control groups, these marker panels remarkably improved the sensitivity (1.00, CI: 0.95-1) with a specificity of 0.80 (CI: 0.67–0.90)	[66]
		miR-1246 and miR-4644	Saliva	N = 12	The relative expression ratios of miR1246 and miR4644 were significantly higher in the cancer group than these ratios in the control group with AUC of 0.814 and 0.763, respectively.	[67]
		miR-21 and miR-155	Pancreatic juice	N = 27	Relative levels of both ex-miR-21 and ex-miR-155 in EVs were significantly higher in PDAC patients compared with chronic CP patients	[68]
mRNA		GPC-1 mRNA	Serum	Stage I- II n = 86, stage III-IV n = 32	GPC1 mRNA was enriched in pancreatic cancer patients and could be used to classify patients with healthy donors with AUC of 1.00 and sensitivity and specificity of 100%	[69]
		CK18 and CD63 RNA	Plasma	N = 57	Biomarker panel consisted of miRNA, mRNA, CA19-9, and cell free DNA for PDAC diagnosis achieved an accuracy of92% (95% CI, 86-96%), with sensitivity of 88% (95% CI, 76-95%) and specificity of 95% (95% CI, 88-99%)	[70]
		Apbb1ip, Aspn, BCO31781, Daf2, Foxp1, Gng2,and Incenp	Saliva	N = 22 (mouse)	The 7 biomarkers were significantly elevated in in pancreatic cancer-bearing mouse saliva when compared with control saliva (P < 0.05)	[71]
lncRNA		Sox2ot	Plasma	N = 61	Sox2ot in sEVs was significantly associated with cancer stages (P = 0.014) and was also related to lymphatic or vascular invasion, showing potential as prognosis marker	[72]
		HULC	Serum	N = 20	The expression of HULC in sEVs was significantly higher in PDAC patients than in healthy individuals or IPMN patients with AUC of 0.92	[73]
		Malat-1 and CRNDE	Serum	N = 2	Significantly Higher expression levels of Malat-1 and CRNDE in PDAC-derived sEVs than in healthy donors with P of 0.018 and 0.028	[74]
		FGA, KRT19, HIST1H2BK, TIH2,MARCH2, CLDN1, MAL2 and TIMP1	Plasma	N = 284	The signature of a combination of 8 RNAs in sEVs showed high accuracy in PDAC detection with AUC of 0.960, 0.950 and 0.936 in the training, internal validation and external validation cohort, respectively.	[75]
circRNAs		Circ-IARS	Plasma	N = 40	Circ-IARS expression was up-regulated in pancreatic cancer tissues and in EVs of patients with metastatic disease with P of 0.015 and 0.002, respectively	[76]
		Circ-PDE8A	Plasma	N = 60	High levels of circ-PDE8A were associated with tumor progression and prognosis	[77]

miRNAs are small, noncoding RNAs of ~ 19–24 nucleotide length and regulate about 70% of mRNA transcripts in humans, playing vital roles in a variety of cellular processes such as cancer development. [78, 79] Oncogenic and cancer-suppressor miRNAs in sEVs may be of high diagnostic/prognostic value in PDAC because of their differential expression between cancer cells and normal cells. For instance, Takahasi et al. profiled the expression of miRNAs of sEVs from plasma of 50 PDAC patients and 20 healthy volunteers using real-time quantitative reverse transcription (qRT-PCR) and found that miR-451a showed higher upregulation in the patients and was associated with PDAC stages.^60^ In another study, Xu et al. analyzed miRNA expression in sEVs from plasma of 15 PDAC patients and 15 healthy people using qRT-PCR and discovered that both miR-196a and miR-1246 were increased in the serum-derived sEVs of PDAC patients, as compared to the controls.[58] Interestingly, few studies indicated that some miRNAs might decrease in patients, which can also be used as biomarkers for PDAC diagnosis/prognosis. For example, Lai et al. compared the miRNA levels in the sEVs from healthy people and PDAC patients. They discovered that PDAC patients expressed higher levels of some miRNAs such as miR-21, miR-10b, miR-30c, and miR-181a and lower levels of some miRNAs such as miR-let7a and miR-122 in sEVs from serum, which could be used to differentiate healthy controls and PDAC patients.[13] According to ROC curves, miR-10b, -21, -30c, -181a, and -let7a in sEVs all have 100% sensitivity and specificity in detecting PDAC from normal controls, while miR-106b and − 483 failed to distinguish these two groups. Furthermore, another 3 serum-derived sEV RNAs (ANLN, ITGA6, and KRT18) were recently reported lower expression in PDAC than benign pancreatic diseases or healthy controls, while sEV RNA MMP9 showed relatively higher level in advanced PDAC patients than in early stage patients.[80]To further improve the detection accuracy and promote the clinical application of sEVs for PDAC diagnosis, the combination of miRNAs and other molecules such as proteins have been applied for PDAC diagnosis. For instance, a study indicated a combination of proteins (CD44v6, Tspan8, EpCAM, MET, and CD104) and miRNAs (miR-1246, miR-4644, miR-3976, and miR-4306) in serum-derived sEVs could improve the diagnostic accuracy of PDAC with a sensitivity of 1.00 (CI: 0.95–1) and specificity of 0.80 (CI: 0.67–0.90).[66] Results of these studies demonstrated that these miRNAs could serve as diagnostic and prognostic indicators for PDAC.

mRNAs in sEVs have also been reported to be PDAC biomarkers.[81] In a study by Hu et al., glypican-1 (GPC-1) mRNA in sEVs from serum with an AUC of 1.0 or 100% specificity and 100% sensitivity in each stage of PDAC were identified by a biochip, distinguishing patients with early- and late-stage PDAC from healthy donors and patients with benign pancreatic disease.[69] IncRNAs are nonprotein-coding RNAs with more than 200 nucleotides, playing an important role in regulation of gene expression and pathogenesis in cancers.[82] Kenji et al. analyzed lncRNA “highly upregulated in liver cancer (HULC)” expression from serum sEVs of 20 PDAC patients, 22 intraductal papillary mucinous neoplasm (IPMN) patents and 21 heathy individuals, showing significantly higher HULC level in PDAC patients than others.[73] Apart from above RNAs, circRNAs, which was recognized as a novel class of highly stable noncoding RNA species, may also act as a biomarker for PDAC diagnosis. [83] Specifically, Li et al. found an elevated expression level of circRNAs such as circPDE8A and circIARS, in tumor tissues and sEVs from the plasma of PDAC patients. The overexpression of the circRNAs in sEVs was speculated to contribute to tumor invasion and metastasis.[76, 77].

All in all, RNAs in PDAC-derived sEVs showed promising performance in PDAC diagnosis/prognosis. However, there is still some disputes. John et al. found most individual sEVs isolated by ultracentrifugation contained biologically insufficient quantities of miRNAs, accompanied by a small proportion of free miRNAs from plasma, making them unlikely to serve as miRNA-based communication vehicles.[84] Currently, there are no RNA-based detection method for PDAC in clinic due to various reasons, such as heterogeneous nature of sEVs, difficulty in pure RNA extraction from sEVs and lack of validated RNA biomarkers. To realize their potential value as biomarkers for clinical application, more efforts are needed to discover new RNA biomarkers, develop highly sensitive and specific detection techniques and evaluate their significance in PDAC diagnosis/prognosis. Excitedly, two clinical trials (NCT03821909 and NCT04636788) were started to investigate the diagnostic and prognostic values of small RNA biomarkers in sEVs for PDAC diagnosis in August of 2018 in affiliated Nanjing drum tower hospital of Nanjing University medical school and in November of 2020 in Tongji hospital (Tongji medical college), respectively.

### DNAs

Owing to their ability to carry information regarding cancer-associated mutations, DNAs in cancer-derived sEVs are of great value as a diagnostic/prognosis tool.[85, 86] Thus, detection of DNA mutations in sEVs from PDAC patients can be potentially used for PDAC diagnosis/prognosis.

Wan et al. developed a device utilizing sEV size-matched silica nanostructures and a surface-conjugated lipid nanoprobe to enrich sEVs from the plasma of 3 PDAC patients and 2 healthy controls. They confirmed that the concentration of DNA with the KRAS mutation was higher in patients than controls. [87] In a study with higher number of PDAC patient cohorts by Allenson et al., sEV KRAS mutations were detected in 66.7% (22/33), 80% (12/15), and 85% (17/20) of localized, locally advanced, and metastatic PDAC, respectively, and in 7.4% (4/54) of healthy controls in the discovery cohort. In the validation cohort of 121 individuals, mutant KRAS DNA in sEVs was detected in 43.6% (17/39) of early-stage PDAC patients and 20% (17/82) of healthy controls. [88] Furthermore, Vincent et al. also reported that KRAS mutant allele fraction (MAF) from sEV DNA provides both predictive and prognostic information for PDAC based on data from 123 serial blood samples. [89]

Multiple DNA mutations in cancer-derived sEVs were also explored for PDAC diagnosis/prognosis. F.A. San et al. identified multiple DNA mutations from plasma-derived sEVs of 2 PDAC patients and found NOTCH1 and BRCA2 in the samples. [90] In another study with bigger cohort, Yang et al. investigated the potential clinical utility of sEV DNA from the serum of 114 healthy subjects, 7 IPMN patients, 9 CP patients, 48 PDAC patients and 12 other patients (diseases such as autoimmune pancreatitis, common bile duct cancer) for identification of both KRAS^G12D^ and TP53^R273H^ mutations.[91] They found that sEV DNA harbors KRAS^G12D^ mutation in 39.6% of cases, and TP53^R273H^ mutation in 4.2% of cases of PDAC patients, while 2.6% of healthy subjects presented with KRAS^G12D^ mutation and none with TP53R^273H^ mutation in the sEVs, indicating the strong potential of circulating sEV DNA for cancer diagnosis/prognosis.

All these studies demonstrate the value of DNA mutations in sEVs as potential biomarkers for PDAC diagnosis/prognosis. Considering that some healthy people also had these DNA mutations in sEVs, this approach may also be used to predict the development of PDAC. However, it should be noted that a DNA mutation does not indicate the presence or prognosis of cancer. In addition, DNA methylation has also been found to play an important role in the initiation and progression of many tumors, such as gastric cancer.[92] The role of DNA methylation in PDAC diagnosis/prognosis will need further investigations.

### Proteins

sEVs contain a great number of cytosolic proteins (enzymes, cytokines, apoptotic proteins, oncoproteins, etc.) and surface proteins (adhesion molecules, integrins, tetraspanins, etc.). According to the current version of sEV content database, 9769 proteins have been identified associated with sEVs and 745 of them are relevant to pancreatic cancer.[93] Analysis of these sEV proteins is a powerful tool for PDAC diagnosis/prognosis, due to their unique characteristics compared with traditional serological markers. For example, sEV proteins show higher stability, as they are protected by the lipid bilayer from degradation by extracellular proteases and enzymes.[94] Notably, abundant cancer-associated proteins have been identified in PDAC-derived sEVs, and their types and expression levels are strongly correlated with the presence and progression of PDAC.[95] The aberrantly expressed proteins in PDAC-derived sEVs distinguish them from sEVs of healthy donors or patients with other diseases, and make them a novel means to identify PDAC.[96–98] Table2 summarizes the sEV protein biomarkers for PDAC diagnosis/prognosis.

**Table 2:** sEV protein biomarkers for PDAC diagnosis/prognosis

Biomarkers	Sources	Patient numbers	Discoveries and diagnostic performance	Ref.
GPC-1	Plasma	N = 27	High GPC-1 in sEVs may be able to determine PDAC tumor size and disease burden. AUC of 0.59 was achieved for PDAC detection.	[99]
MIF	Plasma	N = 40	MIF was highly expressed in sEVs from PDAC patients (PDAC patients without liver metastasis vs. healthy controls P < 0.01)	[83]
EpCAM	Plasma	N = 19	PDAC patients had a high level of EpCAM in sEVs, and the level changed during palliative chemotherapy treatment	[100]
EphA2	Plasma	N = 49	EphA2 in sEVs could distinguish pancreatic cancer patients from pancreatitis patients and healthy subjects with AUC of 0.93–0.96	[101]
KRAS^mut^, P53^mut^	Plasma	Stage I n = 16	Mutant proteins KRAS^mut^ and/or P53^mut^ were detected in 15 of the 16 early stage PDAC patients	[102]
EGFR, CA19-9	Plasma	N = 5	More abundant of EGFR (5 fold) and CA19-9 (15 fold) enriched sEVs in PDAC patients than healthy donors	[103]
EGFR, EpCAM, MUC1, GPC1, WNT2	Plasma	N = 22	The five-marker signature yielded a more accurate diagnosis of PDAC than CA19-9 and a single sEV biomarker with sensitivity of 86% (CI, 65 to 97%) and a specificity of 81% (CI, 58 to 95%) in prospective cohort	[95]
GPC-1, CD63	Plasma, serum	N = 20	Twenty PDAC patient samples could be distinguished from 11 healthy subjects with 99% sensitivity and 82% specificity	[104]
GPC-1, EpCAM, CD44V6	Plasma	N = 9	The PDAC EV signature of the three protein biomarkers could be used for PDAC diagnosis with AUC of 1.000 (95% CI: 84.6–100%) and showed strong correlation with cancer stages	[105]
GPC-1	Serum	N = 190	GPC-1 in sEVs showed higher level in PDAC patients than healthy donors with P < 0.0001	[96, 106, 107]
c-Met	Serum	N = 55	Diagnostic test based on c-Met in sEVs resulted in a sensitivity of 70%, a specificity of 85%	[108]
CKAP4	Serum	N = 47	The CKAP4 levels in sEVs were higher in patients with PDAC than healthy control individuals	[109]
ANXA6	Serum	N = 108	ANXA6 level in sEVs could be used to diagnose PDAC patients with AUC of 0.979 and improved sensitivity and specificity	[110]
ZIP4	Serum	N = 24	The level of ZIP4 in sEVs showed promising diagnostic efficacy between PDAC and control group with AUC of 0.893	[97]
ADAM8	Serum	N = 5	ADAM8 in EVs from PDAC patients or precursor lesions had significantly higher expression when compared to healthy individuals with P < 0.0001or P = 0.0139, respectively	[64]
CD41, CD61, CD63	Serum	N = 39	The levels of CD41, CD61 and CD63 in sEVs increased in PDAC patients then healthy donors with AUC of 0.678, 0.652 and 0.846, respectively	[111]
CD44v6, C1QBP	Serum	N = 142	Highly expressed CD44v6 and C1QBP in sEVs were promising biomarkers for predicting prognosis and liver metastasis in patients with PDAC	[112]
LRG-1, GPC-1	Serum	N = 15	Combination of LRG-1 and GPC-1 positive sEVs could improve the diagnostic accuracy of PDAC with AUC of 0.95, even for the early stage PDAC.	[113]
Integrin α6	Blood	N/A	The expression of Integrin α6 in sEVs from blood of PDAC patients significantly decreased after surgery and increased several months before clinical recurrence	[114]
Mucin-4, Mucin-5AC, Mucin-6, Mucin-16, etc.	Pancreatic duct fluid	N = 4	Unique proteins were detected exclusively in sEVs from Pancreatic duct fluid by mass spectroscopy (MS)	[115]
Combination of 35 proteins	Pancreatic duct fluid	N = 13	Pancreatic duct fluid proteins were potential biomarkers of patients with different pancreatic diagnoses	[116]

In 2015, Melo et al. reported that the level of GPC-1 on circulating blood sEVs was significantly higher in PDAC patients compared to healthy people, with the sensitivity and specificity of GPC-1 detection both at 100% for PDAC diagnosis.[96] In another study, Buscail et al. modified magnetic beads with CD63 antibody to collect PDAC-derived sEVs from the blood and detected GPC-1 positive sEVs by flow cytometry with sensitivity of 64% and specificity of 90%.[117] Furthermore, Liang et al. found that ephrin type-A receptor 2 (EphA2), which expressed on the surface of sEVs from plasma, could be used to distinguish PDAC patients and healthy subjects.[101] Mutant proteins can also be biomarkers for PDAC diagnosis. In a recent study, Scott et al. found the mutant proteins KRAS^mut^ and/or P53^mut^ were positive in plasma-derived sEVs from 15 to 16 stage I PDAC patients using single-sEV analysis technique, showing potential of mutant sEV proteins for early stage cancer diagnosis.[102].

Different proteins played different roles in biologic functions, thus simultaneous detection of multiple proteins on/in sEVs may provide much richer information than each one alone, which may more accurately reflect a molecular signature of the cell from which they originate, comparing to the presence of only one biomarker.[50] For example, Yang et al. used a multiplexed plasmonic assay to analyze circulating cancer-derived sEV biomarkers in the training cohort involving 22 PDAC patients and 10 healthy donors. [95] They found that the five surface membrane proteins (EGFR, EPCAM, MUC1, GPC1, and WNT2) showed relatively different individual expression levels in each subject. The authors also found that GPC-1 had a sensitivity of 55% and a specificity of 60%, whereas the PDAC sEV signature of the five surface proteins showed a sensitivity of 100% and a specificity of 100%. In a more recent study, Juan et al. used bead-based multiplex immunoassay kits to test the isolated sEVs from plasma of stage I-II PDAC patients and healthy donors and found the six protein biomarkers could identify the PDAC with AUC of 0.997 in the training cohort (controls, n = 146; PDAC cases, n = 33) and AUC of 0.978 in the validation cohort (controls, n = 139; PDAC cases, n = 35).[118] The PDAC EV protein signature of sEVs offered higher sensitivity, specificity, and accuracy than the single sEV protein biomarker.

Apart from the membrane proteins on sEVs, proteins inside the sEVs can also serve as biomarkers. Zheng et al. identified the protein complement of sEVs from pancreatic duct fluid of patients with PDAC (n = 13), IPMN (n = 8) and benign pancreatic diseases (n = 5) using mass spectroscopy, and validated the expression by immunohistochemistry. Among all the proteins, the top 35 proteins were significantly associated with PDAC, showing strong potential to be PDAC diagnosis biomarkers.[116] Hiroyuki et al. detected the proteins of sEVs from endoscopic ultrasound-fine needle aspiration (EUS-FNA) biopsy PDAC patients (n = 40) and autoimmune pancreatitis (AIP) patients (n = 6) using nano liquid chromatography tandem-mass spectrometry.[119] 1071 sEV proteins were identified only in PDAC and 153 of them were significantly different between PDAC and AIP, indicating the specific sEV protein barcode of PDAC was promising biomarkers for diagnosis. Mass spectrometry has the potential to greatly promote the proteomics research on cancer-derived sEVs, facilitating the discovery of specific and sensitive protein biomarkers for PDAC diagnosis/prognosis.

### Lipids

Compared to the great attention being paid to nucleic acids and proteins in sEVs, lipids represent other less-explored bioactive molecules abundantly present in sEVs. According to a report of 2019, research into sEV lipidomics accounted for less than 4.3% of the sEV genomics studies, and approximately 5.5% of the sEV proteomics work, indicating low scientific interest in sEV lipid research.[120] However, lipid is one of the most important components in sEVs, playing indispensable roles on the structural and regulatory functions of sEV biogenesis, release, targeting and cellular uptake.[120, 121] Therefore, lipidomic studies of sEVs may be an innovative direction for the discovery of new biomarkers for cancer diagnosis/prognosis.

Currently, lipids of sEVs have been reported to be biomarkers for cancer diagnosis/prognosis in diverse cancers, including prostate cancer,[122] non-small cell lung cancer,[123] and colorectal cancer.[124] So far, very limited studies on lipids in sEVs for PDAC diagnosis/prognosis have been published. Raghava et al. found phosphatidylserine positive sEVs in blood increased significantly in PDAC bearing mouse, suggesting the potential of phosphatidylserine positive sEVs for PDAC detection.[125] Furthermore, Samuel et al. extracted lipid from different PDAC cell-derived sEVs and analyzed them by mass spectroscopy.[126] They found that sEVs derived from AsPC-1, Panc-1, BxPC-3 and HDPE cells all had significantly different lipid expression profiles, including phosphatidylcholine, phosphatidylserine, phosphatidylethanolamine, phosphatidic acid and phosphatidylinositol, which showed potential use for PDAC diagnosis. Charles et al. analyzed 1021 lipid species from sEVs of different pancreatic cancer cell lines (Panc-1, Capan-1, SW-1990, Mia PaCa-2, PPCL-68 and PPCL-46) and normal cell lines (hTERT-HPNE, HPDE-H6c7) dysregulated lipids were observed between cancer-dervied sEVs and normal sEVs, especially these lipid species containing palmitic acid (16:0) and sphingomyelin.[127] In another study by Tao et al., liquid chromatography-data dependent acquisition-mass spectroscopy (LC-DDA-MS) based lipidomic analysis was used to analyze the lipid expression profile in sEVs derived from peripheral blood of 22 PDAC patients and 17 healthy people.[128] The authors found that about 270 lipids were significantly dysregulated between the sEVs of PDAC patients and healthy controls, and 61 significantly dysregulated lipids were further analyzed by LC-MRM-MS to verify the results of lipidomic analysis in sEVs from 24 PDAC patients and 40 healthy donors. They discovered that LysoPC 22:0, PC (P-14:0/22:2) and PE (16:0/18:1) were all associated with tumor stage, CA19-9, CA242 and tumor diameter. These findings revealed that dysregulated lipids in sEVs from PDAC patients show potential as biomarkers for diagnosis/prognosis. The research into the role of lipids in sEVs offers a un-explored avenue for PDAC biomarker discovery.

### Glycans

Glycans consist of oligosaccharides linked glycosidically to proteins, lipids and proteoglycans which are displayed on the exterior surfaces of cells and sEVs. They can be classified into different types of glycans. Specifically, they can be divided into N-linked glycans (attached to the Asn of glycoproteins in a particular Asp-X-Ser/Thr sequon), O-linked glycans (attached to the Ser or Thr of glycoproteins and predominate on mucins), glycosaminoglycans (attached to Ser residues of proteoglycans), and glycolipids (attached variously to lipids).[129] Different types of glycans and their receptor binding lead to different physical properties and cellular functions which attribute to the development and progression of cancer and other diseases.[130, 131] Moreover, glycans play significant roles in intracellular interactions that depend on cellular conditions and the onset of diseases such as PDAC. [132] Therefore, glycans on PDAC-derived sEVs may also have the potential to be a useful biomarker for PDAC diagnosis/prognosis.

To study the roles of glycan changes in pancreatic diseases, Engle et al. inducibly expressed human fucosyltransferase 3 and β-1,3-galactosyltransferase 5 in mice to reconstitute the glycan sialyl-Lewis^a^ (also known as CA19-9) which was a carbohydrate antigen attached to O-glycans on the surface of pancreatic cells. 133They found that CA19-9 expression in mice resulted in rapid and severe pancreatitis with hyperactivation of epidermal growth factor receptor (EGFR) signaling to promote PDAC. Yokose and his colleagues analyzed the differential glycomic profiling of sEVs derived from serum (two cohorts including 117 PDAC patients and 98 normal controls) using lectin microarrays.[134] The glyco-candidates of PDAC-specific sEVs were quantified using a highly-sensitive sEV-counting system, ExoCounter. Quantitative analysis using ExoCounter revealed that the O-glycan-binding lectins, *Agaricus bisporus agglutinin* (ABA) and *Amaranthus caudatus agglutinin* (ACA), positive sEVs were significantly increased in the culture of PDAC cell lines and in the serum of PDAC patients. These specific sEVs with O-glycans recognized by ABA/ACA were elevated in PDAC sera and could act as potential biomarkers in a liquid biopsy for PDAC patient screening. Choi et al. attached lectins with a high and specific affinity for sialic acid or fucose to bifunctional Janus nanoparticles (JNPs) for glycan detection on sEVs by a microfluidic device.[135] sEVs derived from PDAC cell lines and plasma samples of PDAC patients were successfully captured on the lectin-conjugated JNPs. The relatively higher glycan recognition of sEVs from patient plasma by *Sambucus nigra agglutinin* (SNA) and *Aleuria aurantia lectin* (AAL) modified JNPs could thus potentially be used for cancer diagnosis. The abnormal expression of specific glycans on sEVs, their presence in patient serum and plasma, and their possible ability to facilitate metastases, suggests that glycans could contribute to cancer diagnosis/prognosis. To fully exploit glycans for further clinical use, it will be vital to fully profile the glycans of PDAC-derived sEVs and determine how this varies between different cancer stages and healthy people.

Biomolecules such as nucleic acids, proteins, lipids and glycans in PDAC-derived sEVs have all shown the potential to act as biomarkers for PDAC diagnosis/prognosis. RNAs and proteins serve as highly specific biomarker candidates for PDAC detection and represent the most important biomolecules associated with PDAC-derived sEV studies. RNAs affect the cancer associated metabolism and functions. They could be easily extracted and analyzed using the well-developed RNA profiling techniques such as polymerase chain reaction and next-generation sequencing. There are three ongoing clinical trials using RNAs for PDAC detection as mentioned above. sEV proteins include membrane proteins and inner proteins, analysis of which could help us to understand the mechanisms of sEV biogenesis and functions. Membrane proteins are more studied than inner proteins for PDAC detection due to easier sample preparation and rapid and sensitive analysis approaches (e.g. flow cytometry and enzyme-linked immunosorbent assay), which are based on high specific of antibodies. DNA mutations exist in sEVs from both healthy donors and PDAC patients, their role for clinic use need more investigation. sEV glycans and lipids have been explored for cancer diagnosis/prognosis due to the fast development of glycomics and lipidomics in recent years, screening and validation of clinical biomarkers for PDAC diagnosis/prognosis require more efforts. There is no sEV based clinical assays for PDAC detection now. To realize clinical use of these biomolecules, more intensive studies are still required to determine their clinical sensitivity, specificity and accuracy. Moreover, the lack of clinically applicable, reliable detection techniques for these biomolecules makes them difficult to be utilized. These potential problems may be solved by gaining more knowledge about how these biomolecules transport into sEVs, optimizing the extraction procedures and improving the detection techniques of these active molecules, which could eventually lead to the development of a heterogeneous panel of biomarkers for clinical application in PDAC diagnosis/prognosis. Although cancer-derived sEVs as biomarkers for cancer diagnosis/prognosis in patients have been reported in a flurry of papers, very few clinical diagnostic/prognostic assays are implemented. To date, two sEV-based clinical assays have reached the stage of clinical validation and one assay called ExoDx Prostate (IntelliScore) has been launched onto the market in United States since 2016.[136, 137] Briefly, the ClarityDx™ System (NCT03957252) is used for prostate cancer diagnosis by detecting GHSR (ghrelin receptor), PSMA (prostate-specific membrane antigen) and polysialic acid in blood-derived sEVs by micro flow cytometry. Sentinel™ PCC4 test (NCT04100811, NCT04661176) is used for prostate cancer by detecting 442 small non-coding RNAs (sncRNAs) in urinary sEVs. Another test Sentinel™ (NCT04155359) detecting 280 sncRNAs is used for bladder cancer diagnosis. The commercial available assay ExoDx Prostate (IntelliScore) (NCT03235687, NCT03031418, NCT04720599, NCT02702856) is based on the detection of three RNAs (PCA3, SPDEF, ERG) in urinary sEVs from prostate cancer patients by quantitative reverse transcription-polymerase chain reaction (RT-PCR).

## Cancer therapy

The exploration of cancer-derived sEVs for therapeutic purposes is still in its infancy due to the risk of endogenous cargo molecules which may activate pathological pathways.[138] Over the past few years, several cancer-derived sEV based therapeutic approaches have been developed, including inhibition of cancer-derived sEV formation or secretion, using cancer-derived sEVs as a potential drug carrier for target therapy and for immunotherapy. As compared to other therapeutic strategies, cancer-derived sEV based therapies are likely to have targetable ability, high stability, cross biological barrier ability and low toxic side effects.[139] All of these characteristics support the potential application of cancer-derived sEVs in cancer treatment. In this section, we will mainly review the advances in therapeutic strategies using cancer-derived sEVs for PDAC treatment (Table3).

**Table 3:** Cancer-derived sEV based strategies for PDAC treatment

Strategies	sEVs involved	Drugs	Therapeutic performance	Ref.
Inhibition of cancer-derived sEV formation or secretion	CAF-derived sEVs	GW4869	GW4869 treated CAF decreased the release of sEVs and reduced the survival of epithelial cells	[140]
	CAF tumor organoid-derived sEVs	Climbazole, imipramine	Climbazole and imipramine prevented the release of PDAC-derived sEVs and inhibited the growth of organoids and chemoresistance	[141]
	PDAC-derived sEVs (Panc-1, MiaPaCa-2, PSN-1)	RAB27B siRNA	Downregulated miR-155 inhibited the release of cancer-derived sEVs and reduced the GEM resistance	[142]
	CAF-derived sEVs	GW4869	Suppression of CAF-derived sEV secretion could reduce these PTEN targeting miRNAs and restore the PTEN expression	[143]
	Pan02-derived sEVs	Short hairpin RNAs	Knocking down of overexpressed genes ITGβ4 or ITGβ5 remarkably reduced the metastatic ability of cancer cells	[144]
	PDAC-derived sEVs (Panc-1, MiaPaCa-2, etc.)	GW4869, MEK inhibitor	Blocking of VEFG-C could inhibit PDAC early dissemination and cancer malignancy	[145]
Cancer-derived sEVs as drug carrier vesicles	Panc-1-derived sEVs	GEM	Tumor growth was suppressed treated with GEM loaded sEVs in mice model	[146]
	Melanoma cell-derived sEVs	Survivin T34A	Survivin T34A loaded sEVs restored GEM sensitivity to PDAC cell lines and induced a significant increase in apoptotic cancer cell deaths	[147]
	Panc-1-derived sEVs	PTX	RGD modified sEVs showed good affinity for αvβ3 on pancreatic cancer cells and improved the tumor cell targeting ability.	[148]
	Panc-1-derived sEVs	siRNA (siPAK4)	The siPAK4 loaded sEVs induced significant apoptosis of tissue and prolonged survival of PDAC bearing mice	[149]
	TAS-derived sEVs	miR-145	The miR-145 in TAS-derived suppressed the PDAC development	[150]
Using cancer-derived sEVs in immunotherapy	Panc-1-derived sEVs	Immune activating proteins in sEVs	PDAC-derived sEV lysates increased the tumor-killing capacity of DCs/CIKs towards PDAC cells	[151]
	Human pancreas carcinoma cell-derived sEVs	HSP70	HSP70 in sEVs stimulated NK cell migration and caused cytotoxicity against cancer cells	[152]
	Rat PDAC-derived sEVs	N/A	PDAC-derived sEVs supported leukocyte effector functions by strengthening NK and cytotoxic T cell activity	[153]
	PDAC-derived sEVs	SEB	PDAC-derived sEVs loaded with T cell immune stimulator SEB could significantly induce cancer cell apoptosis	[154]
	Immunogenically dying tumor cell-derived sEVs	CCL22 siRNA	CCL22 siRNA loaded in MART-1 peptide modified sEVs could enhance antitumor immune response	[155]
	PDAC-derived sEVs (Panc02)	GTPase Rab11	Inhibition of saliva sEVs could lose their ability to inhibit NK cells	[156]

### Inhibition of cancer-derived sEV formation and secretion

Cancer-derived sEVs play key roles in PDAC progression and in the onset of cancer drug resistance.[35] It has also been reported that cancer-derived sEVs can transport epidermal growth factors to macrophages, thus interfering with the innate immune system’s function.[157] These cancer-derived sEVs not only promoted the cancer development, but also affected the immune system and drug resistance in order to protect the cancer cells. Thus, inhibiting the formation and release of cancer-derived sEVs may delay cancer progression and improve cancer treatment. A growing number of studies have indicated that disrupting the signaling pathway of cancer-derived sEVs was able to block the formation and secretion of cancer-derived sEVs, thus inhibiting tumor growth and metastasis in cancers.[158–161] As aforementioned, the biogenesis of sEVs occurs inside MVBs, and is driven mainly by two mechanisms: endosomal sorting complexes required for transport (ESCRT)-dependent and ESCRT-independent pathways (Fig.3). Manumycin A is an antibiotic that can inhibit RAS (small GTPases) activation, thus disrupting the biogenesis of sEVs by the ESCRT-dependent pathway. GW4869 is able to suppress neutral sphingomyelinase (nSMase), which is an important enzyme for MVB biogenesis, resulting in the inhibition of sEV formation by the ESCRT-independent pathway.[162] Furthermore, the transportation of MVBs, the fusion between MVBs and cell membrane, and the release of sEVs from MVBs, are regulated by many agents, such as the RAB family (a member of the RAS superfamily of small G proteins) for recruiting cytosolic tethers to MVB membranes, soluble N-ethylmaleimide-sensitive factor attachment protein receptor (SNARE) for MVB and cell membrane fusion, Ca^2+^ for calpain activation, and Rho-associated protein kinases (ROCK) for cytoskeleton re‐organization (Fig.3).[162–164].

Cancer-associated fibroblasts (CAFs) are the dominant components of PDAC tumor bulk. Richard et al. found that CAFs played a critical role in promoting the proliferation of PDAC cells through sEV signaling.[140] While CAFs were intrinsically resistant to gemcitabine (GEM), GEM-treated CAFs significantly increased the release of sEVs, which resulted in elevation of a chemoresistance-inducing factor in recipient epithelial cells and promoted proliferation and drug resistance. The treated GEM exposed CAFs were then treated with sEV release inhibitor GW4869 and the survival of epithelial cells was significantly reduced, indicating an important role of cancer-derived sEVs in chemotherapeutic drug resistance. Weikun et al. built a bio-mimetic 3D co-culture model to integrate the complex tumor organoids and used sEV release inhibitor climbazole and imipramine to treat the PDAC organoids, finding that climbazole and imipramine inhibited the growth of organoids and chemoresistance by preventing the release of CAF tumor organoid-derived sEVs. [141] In another study, Mikamori et al. found GEM resistance in PDAC cells might be relevant to miR-155, which controlled genes required for sEV synthesis.[142] The authors used RAB27B siRNA (siRAB27B) to transfect PDAC cells and downregulated RAB27B effectively. The resultant RAB27B knockdown resulted in the remarkable reduction of the amounts of sEVs and GEM resistance in PDAC cells with or without transfection with pre miR-155. These results indicated that targeted miR-155 therapy and inhibition of cancer-derived sEV secretion may be an effective way to reduce or eliminate GEM resistance and to help improve the therapy of PDAC. PDAC patients normally have altered tumor suppressor phosphatase and tensin homolog (PTEN) and low expression of PTEN may promote PDAC progression.[165, 166] Katherine et al. discovered GEM treated CAFs could release PTEN targeting miRNAs (miR-21, miR-181a, miR-221, miR-222, and miR-92a) and the suppression of CAF-derived sEV secretion with inhibitor GW4869 could reduce these PTEN targeting miRNAs and restore the PTEN expression.[143] However, these strategies involve the inhibition of sEVs being secreted from both healthy cells and cancer cells. Therefore, their use in cancer treatment should be carefully considered and investigated, as sEVs also play an essential role in regulating normal biological functions as well.

**Fig. 4:**
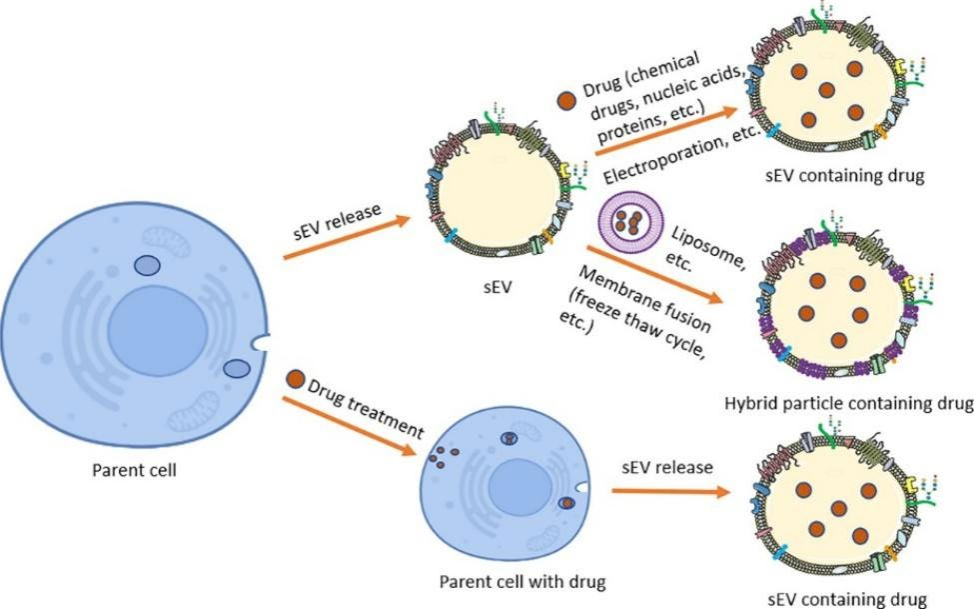
Strategies of using cancer-derived sEVs for drug delivery. Chemotherapy drugs, nucleic acids and/or proteins can be loaded into sEVs by direct or indirect methods

It would be safer to only inhibit or remove the cancer cells-derived sEVs. For example, Hoshino et al. found that ITGβ4 or ITGβ5 genes were overexpressed in pancreatic cancer cell-derived sEVs; knocking down or inactivating these genes dramatically reduced the metastatic ability of pancreatic cancer cells.[144] A more recent study by Capello et al. indicated that PDAC-derived sEVs displayed a wide variety of tumor-associated antigens which could bind circulating autoantibodies.[167] PDAC-derived sEVs exerted a decoy function and possibly attenuated complement-dependent cytotoxicity against PDAC cells, which suggested that it may be possible to inhibit PDAC sEV secretion or selectively eliminate the circulating PDAC-derived sEVs through affinity capture as one way to treat PDAC. One interesting finding is that the inhibition of a specific molecule on cancer-derived sEVs could also improve cancer treatment. Chun-an et al. found vascular endothelial growth factor-C (VEGF-C) on PDAC-derived sEVs promoted lymphangiogenesis and thus blocking of the production of VEFG-C could inhibit PDAC early dissemination and cancer malignancy.[145].

PDAC-derived sEVs have been reported to promote immune suppression, cancer metastasis and chemoresistance in PDAC. The suppression of PDAC sEV secretion or disruption of the signaling pathways is thus an achievable strategy to treat PDAC. To bring these therapeutic approaches to clinical application, it is essential to comprehensively understand the biogenesis of PDAC-derived sEVs as well as their signaling pathways and elucidation of the mechanisms as to how PDAC-derived sEVs contribute to cancer metastasis and chemoresistance.

### Cancer-derived sEVs as drug carrier vesicles

In recent years, researchers have made great progress in the development of sEVs as drug carriers. Compared with liposomes and other nanoparticles, sEVs possess better biocompatibility and are considered as a natural way for drug delivery. Injected sEVs shed from endogenous cells of the body are tolerated with minimal immune reaction and toxicity.[139, 168] The therapeutic cargos can be efficiently delivered into the tumor microenvironment using sEVs since these vesicles display efficient target-homing capabilities and easily penetrate through natural biological barriers due to their small particle size.[139, 168] In addition, sEVs express the integrin-associated transmembrane protein CD47 that protects sEVs from phagocytosis by monocytes and macrophages, thus increasing the sEVs’ half-life.[169] Because of these unique advantages, sEVs have emerged as ideal candidates for the delivery of therapeutic cargos, such as chemotherapy drugs, nucleic acids, and proteins.[170, 171] Therapeutic agents can be loaded into sEVs by direct methods such as incubation, sonication and electroporation, or by indirect methods using a drug to treat parent cells and collecting the released sEVs containing the drugs.[172] A series of clinical trials in phase I and II have demonstrated that sEVs hold strong potential for drug delivery.[173] sEVs from different sources such as mesenchymal stem cells, macrophages, dendritic cells, embryonic kidney cells, cancer cells have been used for drug delivery.[174] For example, Kamerkar et al. loaded short interfering RNA or short hairpin RNA in sEVs from fibroblast-like mesenchymal cells to target oncogenic KRAS^G12D^ in Panc-1 tumor bearing mouse models and moved forward to a Phase-I clinical trial by the M.D. Anderson Cancer Center in January of 2021 (NCT03608631) [169, 175].

Cancer-derived sEVs accumulate preferentially in the tumor tissue via the so-called passive targeting ability, which may be ascribed to the presence of several classes of proteins (e.g. integrins, tetraspanins and cancer-specific antigens) on their surface.[47, 176] For example, Xu et al. investigated the uptake properties of sEVs derived from Panc-1 and other cell lines (B16-F10, HEK-293) and Panc-1-derived sEVs showed significant higher uptake in Panc-1 cells than other types of sEVs in vitro and in vivo (Panc-1 tumor bearing mouse model), demonstrating the passive homing ability of Panc-1 sEVs.[177]Additionally, modification the surface of cancer-derived sEVs with targeting molecules such as ligands, aptamers and antibodies can improve the targeting ability (active targeting ability).[176] Furthermore, some researchers proposed that fusing sEVs with conventional synthetic nanoparticles such as liposomes to form hybrid particles (combination of two unique entities) could achieve the beneficial properties associated with both sEVs and synthetic nanoparticles. Fig.4 illustrates the strategies of using cancer-derived sEVs for drug delivery.

Li et al. loaded GEM into autologous Panc-1 cell-derived sEVs for targeted chemotherapy of PDAC.[146] GEM-loaded sEVs facilitated the cellular drug uptake and significantly suppressed tumor growth in tumor bearing mice; even tumors in several mice disappeared without recurrence after treatment. PDAC treatment response however may be affected by cancer associated macrophages. Cristina et al. demonstrated these cancer associated macrophage-derived sEVs decreased the PDAC cell sensitivity to GEM, which was caused by chitinase 3-like-1 (CHI3L1) and fibronectin (FN1).[178] In order to overcome GEM resistance in PDAC, Aspe JR et al. delivered survivin T34A by melanoma cell-derived sEVs to restore GEM sensitivity to PDAC cell lines, inducing a significant increase in apoptotic cancer cell deaths, compared with using GEM alone.[147] More recently, Hasan et al. engineered the surface of PDAC (Panc-1)-derived sEVs by conjugating with functional ligand RGD (peptide composed of several repetitions of Arg-Gly-Asp) and magnetic nanoparticles, then loaded PTX into the modified sEVs to treat xenograft mice bearing Panc-1 tumor.[148] RGD showed good affinity for αvβ3 that was highly expressed in pancreatic cancer cells, thus RGD modification on sEVs could improve the tumor cell targeting ability. The authors also found one important molecule integrin β3, which was expressed in Panc-1 cells and Panc-1 sEVs, involved in the home-driving properties of Panc-1 sEVs to their parent cancer cells. This therapeutic formulation based on the passive and active targeting ability of cancer-derived sEVs could efficiently deliver the drug to the cancer cells and significantly reduced the tumor size when comparing with the control groups. In addition to chemical drugs, RNA drugs have also been shown to be highly effective for PDAC treatment. Xu et al. encapsulated siRNA (siPAK4) into PDAC-derived sEVs by electroporation for PDAC treatment in a mouse model. The siPAK4 induced significant apoptosis of tissue and prolonged survival of PDAC bearing mice with minimal toxicity.[149] Another study by Song et al. found selective packaging of miRNAs into EVs could lead to enrichment of stromal specific miR-145 in sEVs from tumor-associated stroma (TAS). The TAS-derived miR-145 was able to be delivered into PDAC cells by the sEVs and suppressed the cancer development.[150] sEVs have also been engineered to enhance the loading ability of therapeutic cargo or to improve the targeting ability for treatment of other cancers [179], [180].

Collectively, these results indicated an interesting potential utility of cancer-derived sEVs as candidates for different therapeutic agent delivery that could be used to develop innovative treatment strategies for PDAC. However, it should be noted that the endogenous cargos such as oncogenes in cancer-derived sEVs may be associated with cancer progression and migration,[174] thus their potential safety concerns still remain. Several clinical trials have been conducted using cancer-derived sEVs and no serious safety issues were reported in these studies [181–183]. For example, Guo et al. used autologous tumor cell-derived nanoparticles loaded with a chemotherapeutic drug (methotrexate) to treat lung cancer patients with pleural effusions (4 females and 8 males).[182] Six mild adverse events (grades 1 to 2) were observed through the treatments and no acute autoimmune reactions were recorded. In another clinic trial (NCT01854866), Huang et al. investigated the safety and effectiveness of cancer-derived sEVs loaded with chemotherapeutic drugs for malignant ascites and pleural effusion treatment and reported no typical side effects.[184] Furthermore, the development of new techniques that remove the harmful contents in cancer-derived sEVs or preparation of sEV mimetics might helpful to improve the safety. The drug loading capacity of cancer-derived sEVs is also one of the obstacles. Thus a few studies reported the fused sEVs with other nanoparticles such as liposomes or synthesis of sEV mimetics to enhance the loading capacity.[45] Moreover, the improvement of the drug loading method such as electroporation, saponin-assisted loading and extrusion could further increase the loading capacity of sEVs.[185] Another major problem for drug loading using cancer-derived sEVs is their low yield. The effective sEV dose is 10–500µg sEV proteins to each mouse or 0.5–1.4 × 10^11^ sEVs to each patient in one clinic trial.[186–188] Thus, the strategies to obtain large-scale clinical-grade sEVs are required for in vitro basic research, in vivo preclinical animal models, and clinical trials.

### Using cancer-derived sEVs in immunotherapy

**Fig. 5:**
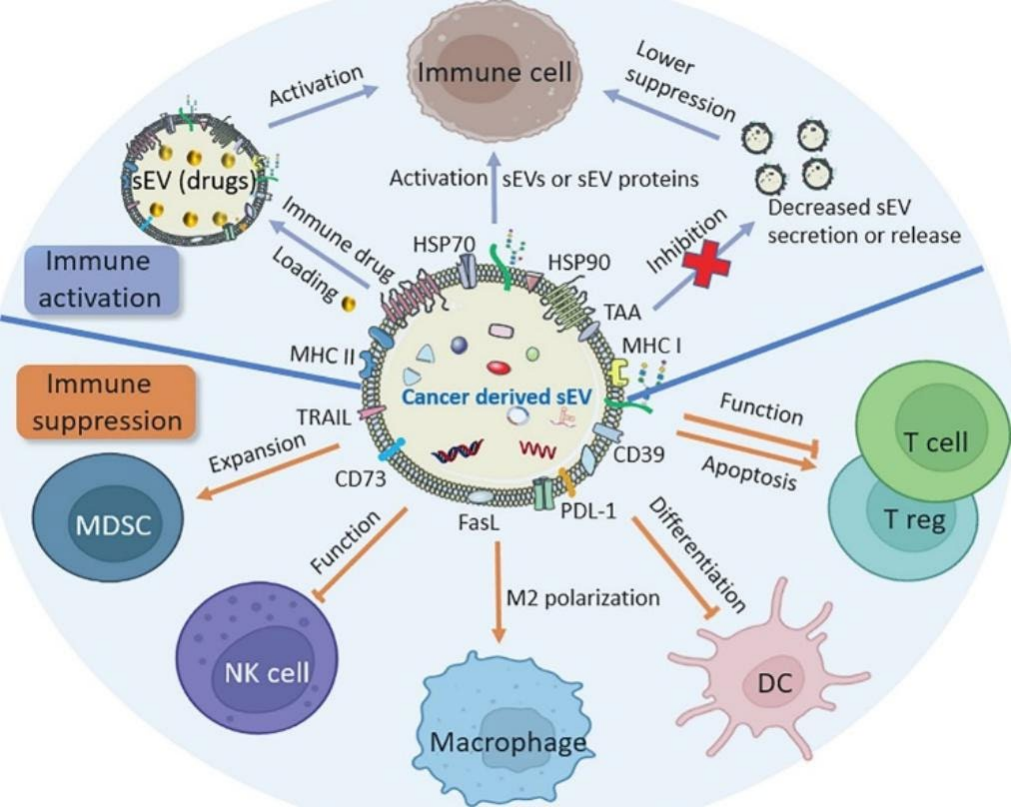
Effects of cancer-derived sEVs on immune cells and therapeutic strategies for cancer treatment by immunotherapy. Cancer-derived sEVs contain immunosuppressive and immunostimulatory molecules, which can be used to activate immune cells. Loading immune drugs into sEVs or inhibition of cancer-derived sEV secretion are two other strategies for immunotherapy

Antitumor immunity is triggered when immune effector cells such as natural killer (NK) cells are activated. sEVs derived from immune cells, such as NK cells, dendritic cells (DCs) and bone marrow mesenchymal stem cells (BM-MSC), can inhibit PDAC progression.[189, 190]. Similarly, sEVs derived from cancer cells carrying immunosuppressive molecules such as Fas ligand (FasL), TNF-related apoptosis-inducing ligand (TRAIL), programmed death-ligand 1 (PD-L1), and enzymes engaged in the adenosine pathway (CD39 and D73) were found to mediate the functions of immune cells, such as activation of regulatory T-cells (T regs), DCs, macrophages and immature myeloid-derived suppressor cells (MDSCs) (Fig.5**)**, thus playing important roles in the establishment of an immunosuppressive microenvironment.[191–193]. For example, these cancer-derived sEVs caused the apoptosis of CD8 + T cells, which is critical for immune defense against cancer, by activating death receptor pathways.[194] Although cancer-derived sEVs have thus been shown to have an immunosuppressive effect on various immune cells, some studies also indicate these sEVs may contain immunostimulatory factors (hear shock protein 70/90 (HSP70/90), major histocompatibility complex class I/II (MHC I/II), tumor-associated antigens (TAAs), etc.) and serve as anti-tumor agonists or vaccines to increase cancer antigen recognition and promote tumor clearance.[152, 195,196,197] Therefore, cancer-derived sEVs are considered to be valuable agents for the treatment of cancer. Although current progress of using cancer-derived sEVs for PDAC immunotherapy is very limited, it is an emerging field for study. Fig. 5 illustrates the effect of cancer-derived sEVs on immune cells and possible therapeutic strategies for cancer treatment by immunotherapy.

Que et al. investigated the proteins of PDAC-derived sEVs and their effect on immune cells DCs/ cytokine-induced killer cells (CIKs) for anti-tumor activity.[151] They isolated sEVs from Panc-1 cell supernatants, then ruptured and ultrafiltered the sEV lysate. 128 proteins, including several immune-activating proteins (attractin, complement C3, C4 and C5, integrin and lactotransferrin), remained in the lysate, while the miRNAs from sEVs were removed. The authors found that these PDAC-derived sEV lysates increased the tumor-killing capacity of DCs/CIKs towards PDAC cells, suggesting that miRNA-depleted cancer-derived sEVs might represent valuable immunotherapeutic tools in PDAC treatment. In line with this study, Gastpar et al. found one protein, HSP70, enriched from PDAC-derived sEVs, stimulated NK cell migration and caused cytotoxicity against cancer cells.[152] Another study discovered that PDAC-derived sEVs, from rats, supported leukocyte effector functions by strengthening NK and cytotoxic T cell activity and showed a minor effect on leukocyte activation. Thus, these cancer-derived sEVs might be used as adjuvant in immunotherapy.[153].

Cancer-derived sEVs can also be used as vectors of immune drugs for cancer immunotherapy. Mahmoodzadeh et al. used PDAC-derived sEVs as carriers to load staphylococcal enterotoxin B (SEB), which was used as a potent immune stimulator for T cell activation.[154] They demonstrated that SEB-loaded sEVs could significantly induce cancer cell apoptosis and no cytotoxic effect was observed in both human kidney embryonic cells and peripheral human white blood cells, indicating that such a strategy might be used to stimulate an immune response against PDAC cells. The authors also investigated the expression of anti-apoptotic genes including *Bax, Bak and Fas* in cells and found the induction of these genes after treatment with SEB-loaded sEVs. In a more recent study, Wenxi et al. modified sEVs derived from immunogenically dying tumor cells with MART-1 peptide (sequence: ELAGIGILTV), which displayed immunogenic properties and was able to expand CD8 + T cells for adoptive T cell transfer.[155] The modified sEVs could enhance antitumor immune response. The authors also loaded CCL22 siRNA into the modified sEVs to suppress Treg expansion, the results demonstrated them as an effective prophylactic vaccine in delaying tumor growth and good adjuvant for chemotherapeutic drugs in PDAC treatment. As cancer-derived sEVs can suppress the functions of some immune cells, the inhibition of these cancer-derived sEV secretion may also improve cancer treatment by immunotherapy. Lau et al. reported that PDAC-derived sEVs from tumor-bearing mice affected the expression of genes in the salivary glands and subsequently induced changed contents of salivary sEVs in vivo.[156] The salivary sEVs from PDAC tumor-bearing mice regulated the phenotype of NK cells, resulting in inhibition of the antitumor cytotoxicity of NK cells. When these saliva sEVs were inhibited, they lost the ability to inhibit NK cells.

Overall, studies using cancer-derived sEVs as potential immunity enhancers against PDAC have so far provided some preliminary but promising results, which encourages further investigations in this field. There has however been some controversy regarding the biological roles of cancer-derived sEVs in immunotherapy, as these sEVs mediate both immunosuppressive or immunostimulatory responses and it is challenging to reconcile these two aspects. The safety concerns of cancer-derived sEVs is another problem and substantial research is required to evaluate this issue.

## Conclusion and perspectives

In summary, PDAC is a highly aggressive and lethal malignancy mostly due to its late-stage presentation and lack of curative therapies. This malignancy is difficult to diagnose/prognose, monitor and treat. Hence, the development of novel diagnostic/prognostic biomarkers and better therapeutic strategies are urgently needed. Cancer-derived sEVs are abundant in both the tumor environment and circulation system. They act as a non-invasive biomarker and provide diverse biological functional information and medical applications in PDAC diagnosis/prognosis and treatment. The biomolecules in/on sEVs including RNAs, mutated DNAs, proteins, lipids and glycans provide the feasibility for developing non-invasive liquid biopsy for low-risk and routine screening for PDAC diagnosis/prognosis. However, it seems unrealistic to detect a single biomarker for accurate PDAC diagnosis/prognosis as a highly sensitive and specific test. Multiplex biomarker detection of sEVs with simple, sensitive and specific assay is a promising area for further development. Researchers should continue to investigate the use of cancer-derived sEVs as biomarkers to detect PDAC. In addition, these cancer-derived sEVs play vital roles in cancer environment, including cancer progression and metastasis, drug resistance, immune regulation, etc., and hold unique properties for cancer treatment such as intrinsic homing ability to tumor tissues, which make them an advantageous mechanism for cancer therapy. Thus new therapeutic approaches based on the understanding of biological functions of these cancer-derived sEVs may facilitate the development of new PDAC treatments. The targeting ability, safety and drug loading efficiency of cancer-derived sEVs should be further explored to enable effective cancer treatment.

To further implement the clinical application of cancer-derived sEVs for PDAC diagnosis/prognosis, there are several issues to be considered: (1) lack of reliable techniques for sEV separation in high-purity homogeneous form; (2) isolation of cancer-derived sEVs from bodily fluids containing healthy sEVs; (3) screening of multiple and reliable biomarkers; (4) development of highly sensitive, specific sEV detection methods. Given that the lipoproteins and other type of EVs have similar particle size with sEVs, it is impossible to enrich high-purity homogeneous sEVs using single traditional isolation method (e.g. ultracentrifugation and size exclusion chromatography). The combination of multiple isolation methods for specific sEV subtype enrichment might be one of the solutions. For example, particles with similar size of sEVs can be enriched using size exclusion chromatography then purified by immunocapture of the specific subtype of sEVs using antibodies. However, the lack of high discriminating cancer biomarker slow down the progress. With the development of multi-omics tools, multiple and reliable biomarker can be screened and used for cancer detection in the near future. In addition, new techniques are still under exploration to improve the sensitivity and specificity for the detection of sEVs for cancer diagnosis/prognosis. The development of new analysis methods such as machine learning are expected to advance the cancer diagnosis/prognosis as well. To progress the development of cancer-derived sEVs in cancer treatment towards clinic application, there are several tasks researchers should pay more attention: (1) large-scale production and isolation approaches for sEVs; (2) technical difficulties in production of clinical-grade sEVs and establishment of corresponding quality control standards; (3) drug loading capacity; (4) cancer cell targeting efficiency; and (5) safety in human trials. Cancer-derived sEVs based therapeutic strategies are still in the primary research stage, there still a long way to go before they can be applied in clinic. For application of cancer-derived sEVs in therapy, it is essential to develop strict good manufacturing practice (GMP) procedures that include all the parameters such as the type of parent cells, the sample volume and isolation conditions (temperature, speed, time, etc.). Corresponding quality control standards should also be developed to release the qualified sEV products and maintain reproducible manufacturing process, including the acceptable range of particle size, concentration and biomarker profiles. Hybrid particles (e.g. sEV and liposome hybrid) or sEV mimetics which carry various payloads may be an interesting area to explore to improve the drug loading capacity. The development of different drug loading techniques such as electroporation could also increase the loading efficiency. As for the cancer cell targeting efficiency and safety of cancer-derived sEVs in human trials, there are quite limited studies being reported. Further collaborative efforts in multiple disciplines are needed to transform cancer-derived sEVs based therapeutic strategies into practical applications for the benefit of cancer patients.

## Data Availability

Not applicable.
